# Postmortem genetic testing in sudden death: clinical and medico-legal implications

**DOI:** 10.1007/s00414-026-03771-8

**Published:** 2026-05-08

**Authors:** María Sabater-Molina, Elisa Nicolas Rocamora, Serena Munteanu, Maria Dolores Fuentes Bermejo, Eduardo Osuna, Maria D. Pérez-Cárceles, Francisco Pastor Quirante, Juan Ramón Gimeno Blanes, Juan Pedro Hernández del Rincón

**Affiliations:** 1 Department of Legal and Forensic Medicine, Regional Campus of International Excellence “Campus Mare Nostrum”, Faculty of Medicine, University of Murcia, Murcia, 30110 Spain; 2 Cardiogenetic Laboratory, Biomedical Research Institute of Murcia (IMIB), Murcia, Spain; 3 European Reference Network for Rare and Low Prevalence Complex Diseases of the Heart (ERN-Guard Heart), Amsterdam, The Netherlands; 4 Institute of Legal Medicine and Forensic Sciences of Murcia, Murcia, Spain; 5 Department of Legal and Forensic Medicine, Biomedical Research Institute (IMIB), Regional Campus of International Excellence “Campus Mare Nostrum”, Faculty of Medicine, University of Murcia, Murcia, 30110 Spain; 6 Pathology Department, Reina Sofia Hospital, Murcia, Spain; 7 Inherited Cardiac Disease Unit (CSUR), Virgen de la Arrixaca University Hospital, Murcia, Spain; 8 Department of Internal Medicine (Cardiology), University of Murcia, Murcia, Spain; 9 Hospital Universitario Virgen de la Arrixaca, Ctra. Murcia-Cartagena s/n, El Palmar, Murcia, 30120 Spain

**Keywords:** Forensic medicine, Sudden death, Clinical autopsy, Legal implications, Postmortem genetic testing

## Abstract

**Background:**

Anatomopathological autopsy and postmortem genetic testing play a crucial role in forensic medicine, particularly in elucidating the causes of sudden death (SD) that remain unexplained by conventional methods. This study explores their value in detecting inherited cardiac conditions with medico-legal and preventive implications.

**Methods:**

From a 15-year forensic cohort, 12 cases of sudden unexpected death in which conventional autopsy was inconclusive or where a hereditary cardiac condition was suspected, were analyzed. Each case underwent histology, toxicology, and targeted next-generation sequencing panels covering genes associated with channelopathies and cardiomyopathies. Variants were classified according to ACMG/AMP guidelines, and family studies were performed when feasible.

**Results:**

Integrated pathological and genetic analysis identified pathogenic or likely pathogenic variants in several cases, notably in RYR2 and CALM2 (channelopathies) and FLNC and PPP1R13L (cardiomyopathies). In these cases, genetic findings confirmed the diagnosis, while variants of uncertain significance were detected in others. Postmortem genetic testing proved essential in cases with structurally normal hearts or sub-diagnostic findings, such as concealed arrhythmogenic cardiomyopathy. Familial cascade testing uncovered additional carriers, enabling targeted surveillance and preventive measures.

**Conclusion:**

Combining pathological autopsy and postmortem genetic testing significantly improves the diagnostic yield in unexplained SD, uncovers hidden hereditary cardiac conditions, and provides critical information for risk assessment in relatives. Beyond clinical implications, these findings contribute to accurate forensic determinations and prevention of miscarriages of justice. Integrating genetic studies into forensic protocols should become standard practice to ensure both scientific rigor and legal fairness.

**Clinical trial registration:**

Not applicable.

## Introduction

Sudden cardiac death (SCD) is defined as an unexpected death due to cardiac causes that occurs within a short time period, generally within one hour of symptom, or within 24 h of last being seen in good health for unwitnessed deaths [1]. It accounts for approximately 15–20% of all deaths in industrialized countries, with an estimated incidence of 1 in 100,000 individuals per year in young people under 35 years of age [2, 3]. SCD has a profound societal impact, particularly when occurring in individuals with no prior symptoms.

In the younger population, the etiological spectrum of SCD differs substantially from that observed in older adults. Inherited cardiac disorders, including cardiomyopathies (hypertrophic cardiomyopathy (HCM), arrhythmogenic cardiomyopathy (ACM), dilated cardiomyopathy (DCM)) and primary arrhythmia syndromes such as long QT syndrome (LQTS), catecholaminergic polymorphic ventricular tachycardia (CPVT), and Brugada syndrome, are frequently implicated [4, 5]. These conditions are often clinically silent and may lack definitive structural abnormalities at conventional autopsy, resulting in a significant proportion of inconclusive forensic investigations [6].

In this setting, post-mortem genetic testing has emerged as a critical adjunct to conventional forensic examination [2, 3, 7]. The identification of a pathogenic genetic variant may substantially modify the interpretation of the cause of death, particularly in cases initially attributed to external triggers such as physical exertion, emotional stress, or minor trauma, which may act as precipitating factors in genetically predisposed individuals [6, 8, 9]. Furthermore, because inherited cardiac diseases follow Mendelian patterns of transmission, genetic findings obtained post-mortem have direct implications for surviving relatives, enabling cascade screening, early diagnosis, and preventive interventions [5, 10]. Despite the increasing adoption of postmortem genetic testing, standardized protocols and their forensic implementation remain heterogeneous, and standardized protocols are still lacking. Ethical considerations, including genetic privacy, data management, and the appropriate communication of results, further complicate its implementation. Addressing these challenges requires standardized procedures, multidisciplinary collaboration, and continuous training of forensic professionals to ensure scientifically rigorous and ethically responsible use of genetic data.

Accordingly, the aim of this study was to evaluate the diagnostic yield and medico-legal impact of integrating systematic pathological examination with post-mortem genetic testing in a selected cohort of sudden unexpected deaths (SUD) investigated in a forensic setting, particularly in cases that were inconclusive after conventional autopsy or in which an inherited cardiac disorder was suspected.

## Materials and methods

### Study population and case selection

All medico-legal autopsies performed at the Institute of Legal Medicine and Forensic Sciences of Murcia between 2009 and 2023 were retrospectively reviewed. During this period, 343 cases fulfilled criteria for SUD and the cause of death was established through conventional autopsy, including comprehensive cardiac examination, histopathological analysis, and toxicological screening. In cases in which a familial inherited cardiac condition was suspected, particularly channelopathies, postmortem genetic testing was also performed as part of the forensic investigation.

From the overall SUD cohort, 12 cases were deliberately selected for inclusion in the present study based on their specific medico-legal relevance. Selection criteria were the absence of a definitive cause of death after conventional autopsy and/or the presence of findings suggestive of an underlying hereditary cardiac disorder with potential implications for medico-legal interpretation. Importantly, these cases were not selected solely on the basis of having undergone genetic testing, but because they represented recurrent and clinically relevant forensic scenarios in which post-mortem genetic testing could play a decisive role in establishing, refining, or excluding the cause of death.

These scenarios included sudden death in children and young individuals with structurally normal hearts, deaths occurring during physical exertion or emotional stress with possible alternative medico-legal interpretations, and cases presenting sub-diagnostic or borderline cardiac findings suggestive of concealed or early-stage inherited cardiomyopathy.

Medical history was systematically investigated through interviews with close relatives and, when feasible, by contacting the decedent’s treating cardiologists.

### Autopsy review

Autopsies were performed according to the guidelines for autopsy investigation of SCD [4]. During external examination or autopsy 5 mL blood from the femoral artery was collected for isolation of genomic DNA. All samples were isolated within 48 h postmortem and stored at −20 °C until further processing. Full autopsy included standardized dissection of the heart, its histological examination and toxicological screening.

### Genetic analysis

DNA was isolated from whole blood using standard protocols. Targeted next-generation sequencing (NGS) was performed using custom gene panels tailored to SCD, cardiomyopathies, and channelopathies (see Annex I). All variants were subsequently confirmed by Sanger sequencing and variant classification was performed according to the 2015 guidelines of the American College of Medical Genetics and Genomics and the Association for Molecular Pathology (ACMG/AMP) [11]. Co-segregation analysis and phenotypic correlation with family history were also considered when available after genetic counselling. Cardiological examination was recommended to first-degree relatives-independent of their participation in this study.

### Ethical approval and informed consent

This work was performed under approval from the local Ethics Committee from University Hospital Virgen de la Arrixaca (2021-10−3-HCUVA). All procedures conducted complied with current ethical and legal standards. The next of kin of decedents provided signed specific informed consent for the genetic study, allowing the use of the samples and collected data for research purposes.

## Results

### Overview of the forensic cohort and case selection

Between 2009 and 2023, a total of 343 medico-legal autopsies of SUD were performed at the Institute of Legal Medicine and Forensic Sciences of Murcia. All cases underwent comprehensive forensic investigation, including complete autopsy, standardized cardiac dissection, histopathological examination, and toxicological analysis. Following this evaluation, cardiomyopathies were established as cause of death in 156 cases (45.5%), with a mean age of 43.4 ± 13.6 years old; 135 (86.5%) males. Structurally normal hearts consistent with inherited channelopathies were identified in 72 cases (21.6%), with a mean age of 39.3 ± 17.1 years and 72.2% males decedents. Ischemic heart disease and other structural cardiac causes accounted for 61 cases (18.3%) (47.6 ± 9.9 years; 88.5% male), whereas 54 (15.7%) cases were attributed to non-cardiac causes (30.1 ± 19.4 years; 66.7% male). A personal or familial history suggestive of inherited cardiac disease was documented in 77 individuals (22.4%) prior to death.

Postmortem genetic testing was undertaken in 119 cases in which an inherited cardiac disorder was suspected. A genetic variant was identified in 93 cases (78.1%). Among these, 39 cases (41.9%) harbored pathogenic or likely pathogenic variants considered sufficient to explain death, while 54 cases carried variants of uncertain significance (VUS).

From this broader cohort, 12 cases were selected for detailed analysis in the present study. Selection was not based solely on the performance of genetic testing, but rather on the coexistence of sub-diagnostic or borderline cardiac findings that were insufficient in isolation to establish a definitive cause of death and/or the presence of circumstances with potential medico-legal implications, such as death during exertion, emotional stress, minor trauma, or occupational activity. In all selected cases, an underlying inherited cardiac disorder was suspected and clarification of the cause and manner of death carried potential legal consequences.

### Pathological findings of case selection

The twelve selected cases demonstrated a heterogeneous spectrum of cardiac findings ranging from structurally normal hearts to definite or suggestive arrhythmogenic cardiomyopathy (ACM), as well as inflammatory and rare structural abnormalities. The main demographic, circumstantial, and pathological characteristics are summarized in Table 1, while the genetic findings are detailed in Table 2.

**Table 1:** Clinical Autopsy Findings in Sudden Cardiac Deaths: Case Summaries

Case ID	Gender	Ethnic group	Age	Circumstances of death	History	Main cardiac pathological findings	Heart weight (g)	ECG findings	Autopsy interpretation
1	F	E	15	Emotional situation	Recurrent syncopes	Structurally normal heart	220	Normal	Inconclusive
2	M	E	9	Physical stress (fair attraction)	No risk factors	Structurally normal heart	150	N/A	Inconclusive
3	F	E	36	Resting at home	No risk factors	Fibro-fatty replacement of RV myocardium	210	N/A	Suggestive of ACM
4	F	E	55	Public space collapse	No risk factors	Extensive fibro-fatty replacement of RV myocardium	480	N/A	Suggestive of ACM
5	M	E	38	Physical exertion (athlete)	Competitive athlete. Prior syncope	Fibro-fatty replacement of RV and LV myocardium	620	Normal	Suggestive of ACM
6	M	E	17	Physical exertion (soccer)	Fatigue/tiredness	Fibro-fatty changes with lymphohistiocytic myocarditis	320	N/A	Suggestive of ACM
7	M	E	43	Sudden collapse	Idiopathic DCM with severely depressed EF	Fibro-fatty degeneration of LV myocardium	495	Abnormal	Suggestive of ACM
8	M	Mo	10	Resting at home	Fatigue/tiredness	LV fibrosis with cardiomyocyte hypertrophy and inflammation	170	N/A	Inconclusive
9	M	Mo	24	Physical confrontation	No risk factors	Cardiac hypertrophy without definitive structural cause	566	N/A	Inconclusive
10	M	E	19	Acute illness	Febrile illness	Severe lymphocytic myocarditis	370	N/A	Inconclusive
11	M	E	38	Working activity	No risk factors	Giant intramural lipoma	350	N/A	Inconclusive
12	M	E	24	Physical exertion (heat exposure)	Recurrent syncopes in stressful situations	Structurally normal heart	390	N/A	Inconclusive

ACM: Arrhythmogenic Cardiomyopathy; DCM: Dilated Cardiomyopathy; EF: Ejection Fraction; EGC: Electrocardiogram;E: European; F: Female; LV: Left Ventricle; M: Male; Mo: Moroccan; N/A: Not available; RV: Right Ventricle

**Table 2:** Genetic Findings in Sudden Cardiac Death Cases Investigated

Case ID	Gene(s) involved	Variant	Variant Classification (ACMG)*	Disease classification	Genes analyzed**	Family screening
1	RYR2	NM_001035.3: c.14311G > T (p.Val4771Phe)	P (PS2, PM1, PM2, PM5, PP2, PP3)	CPVT	1 gene associated with CPVT	Yes
2	CALM2	NM_001743.5: c.404G > T (p.Gly135Val)	P (PS2, PM1, PM2, PP2, PP3)	Long QT	251	Yes
3	JUP	NM_002230: c.103 C > A (p.Pro35Thr)	VUS (PM2, PP3)	RV-ACM	5 desmosomal genes	Yes
4	TXNRD2	NM_006440.4: c.1220G > A (p.Cys407Tyr)	VUS (PM2, PP3)	RV-ACM	218 genes associated with SCD	No
5	GJA5	NM_005266.5: c.365 C > T (p.Ser122Phe)	VUS (PM2)	RV-ACM	218 genes associated with SCD	Yes
6	JUP	NM_021991.2: c.2105G > A (p.Arg702His)	VUS (PM2)	RV-ACM	21 genes associated with ACM	Yes
7	FLNC	NM_001458.4: c.5398G > T (p.Gly1800*)	P (PVS1, PM2, PP1)	LV-ACM	21 genes associated with ACM	Yes
8	PPP1R13L	NM_001142502.1: c.2035 C > T (p.Leu679Phe) (homozygous)	LP (PM3, PM2, PP3)	ACM/DCM	251 genes associated with SCD	Yes
9	LZTR1 LZTR1	NM_006767.3: c.410 C > A (p.Thr137Asn) NM_006767.3: c.614T > C (p.Ile205Thr)	VUS (PM2) VUS (PM2)	Inconclusive (Noonan syndrome)	251 genes associated with SCD	No
10	Negative				21 genes associated with ACM	No
11	Negative				251 genes associated with SCD	No
12	Negative				288 genes associated with SCD	No

CPVT: Catecholaminergic Polymorphic Ventricular Tachycardia; LP: Likely Pathogenic; LV-ACM: Left Ventricle–Arrhythmogenic Cardiomyopathy; P: Pathogenic; SCD: Sudden Cardiac Death; RV-ACM: Right Ventricle–Arrhythmogenic Cardiomyopathy; VUS: Variant of Uncertain Significance. Predicted amino acid change in brackets. *Classification of Genetic Variants According to ACMG Criteria (11). **Annex I. Genetic Panels for the Study of Cardiomyopathies, Arrhythmias, and Sudden Cardiac Death

In three cases (Cases 1, 2 and 12), children and young individuals (27.3 ± 14.5 years old), the heart was structurally normal on macroscopic examination, and extensive histological sampling revealed no evidence of myocardial fibrosis, fatty infiltration, disarray, inflammatory cardiomyopathy, or significant coronary artery disease. These cases fulfilled criteria for autopsy-negative sudden death.

Six cases (Cases 3–8) showed pathological findings within the spectrum of ACM, including right ventricular, left ventricular, or biventricular involvement. The observed changes consisted of focal or diffuse fibrofatty myocardial replacement, sometimes associated with lymphohistiocytic inflammatory infiltrates. In several individuals, these alterations did not fully satisfy established diagnostic criteria for definite ACM and were therefore interpreted as suggestive or sub-diagnostic phenotypes within the ACM spectrum. HCM was excluded based on the absence of myocyte disarray, asymmetric septal hypertrophy, and characteristic architectural abnormalities. Ischaemic heart disease was ruled out by detailed coronary artery examination demonstrating no significant atherosclerotic stenosis or acute plaque complications. When inflammatory infiltrates were prominent, inflammatory cardiomyopathy was considered.

**Fig. 1:**
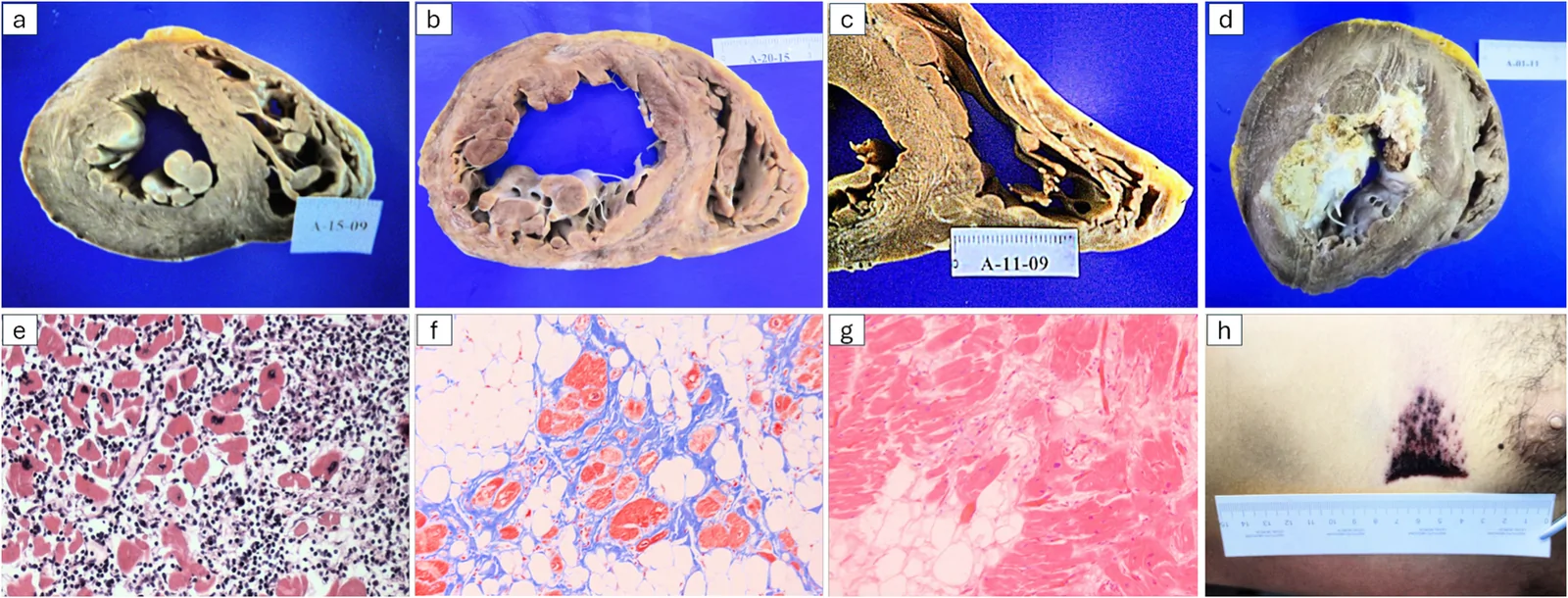
Pathological Findings from Clinical Autopsies of selected cases. (**a**) Structurally normal heart. (**b**) Heart with arrhythmogenic cardiomyopathy predominantly affecting the left ventricle, showing areas of fibro-fatty infiltration. (**c**) Heart with arrhythmogenic cardiomyopathy predominantly affecting the right ventricle, showing myofibrillar degeneration and fibro-fatty replacement. (**d**) Heart with lipoma. (**e**) Histological image of myocarditis, with a lymphohistiocytic inflammatory infiltrate associated with areas of myofibrillar degeneration (H&E stain, 40x). (**f**) Extensive fibro-fatty infiltration in the left ventricle, showing multifocal degeneration (Masson’s trichrome stain, 40x). (**g**) Multifocal fibro-fatty degeneration in the right ventricle, characteristic of arrhythmogenic cardiomyopathy (H&E stain, 40x). (h) Excoriated skin lesion. Insufficient severity to cause death

Case 9 showed marked cardiac hypertrophy with a heart weight of 566 g but lacked definitive histological criteria for hypertrophic cardiomyopathy or ACM, and coronary arteries were free of significant disease. Case 10 demonstrated acute, severe lymphocytic myocarditis involving the conduction system without additional structural abnormalities. Case 11 revealed a giant intramural lipoma measuring approximately 3 cm within the left ventricular wall, without associated fibrosis, inflammation, or coronary pathology. Representative pathological findings are illustrated in Fig. 1.

### Genetic findings in selected cases

Postmortem genetic testing identified pathogenic or likely pathogenic variants in 4 of the 12 selected cases (33.3%), involving genes associated with inherited arrhythmia syndromes or cardiomyopathies (Table 2). In the remaining cases, VUS or negative results were obtained.

In two pediatric cases with structurally normal hearts, genetic testing provided a definitive explanation for death. Case 1 carried a pathogenic de novo variant in RYR2, consistent with catecholaminergic polymorphic ventricular tachycardia, while Case 2 harbored a pathogenic de novo variant in CALM2, associated with severe calmodulinopathy and malignant arrhythmia syndromes. In both cases, the absence of structural abnormalities combined with the presence of well-established disease-causing variants supported classification as primary electrical diseases.

Among cases with pathological findings suggestive of ACM (Cases 3–8), a truncating pathogenic variant in FLNC (p.Gly1800*) was identified in Case 7, supporting a left-dominant ACM phenotype. Case 8 carried a homozygous likely pathogenic variant in PPP1R13L (p.Leu679Phe), compatible with early-onset cardiomyopathy within the ACM/dilated cardiomyopathy spectrum. In these cases, genetic findings reinforced interpretation of structural abnormalities as manifestations of inherited cardiomyopathy rather than isolated or incidental changes.

VUS were identified in five additional cases involving genes related to desmosomal integrity or myocardial structure, including JUP, TXNRD2, GJA5 and LZTR1. Although these variants could not be considered causative in isolation according to ACMG criteria, their identification contributed to the interpretation of borderline pathological findings and supported further clinical correlation and family screening, which was performed in several of these families (Table 2).

Postmortem genetic testing was negative in Cases 10, 11, and 12. In these individuals, death was attributed to non-genetic causes, including acute myocarditis, a rare structural cardiac abnormality in the form of a giant intramural lipoma, and exertional heatstroke, respectively. In these settings, negative genetic results supported exclusion of an inherited cardiac disorder and reinforced the conclusions derived from pathological examination.

### Familial screening and medico-legal implications

Familial cascade screening was performed when feasible and provided clinically relevant information. In Cases 1 and 2, parental testing confirmed de novo status of the variants, allowing reassurance of first-degree relatives. In cases harboring variants within the ACM spectrum, additional carriers were identified among relatives, including clinically affected individuals who subsequently cardiological follow-up. In the family of Case 7, evaluation of 53 relatives identified five carriers of the FLNC truncating variant, two of whom fulfilled diagnostic criteria for cardiomyopathy and three who exhibited non-diagnostic abnormalities, while the remaining relatives were wild-type. In Case 8, heterozygous carriage of the PPP1R13L variant was identified in one sibling, whereas the other was non-carrier.

From a medico-legal standpoint, postmortem genetic testing was particularly relevant in deaths occurring under circumstances potentially suggestive of external responsibility. In Case 9, sudden death during a physical confrontation with only minor external injuries required exclusion of traumatic or homicidal mechanisms; integration of pathological and genetic findings supported a natural cardiac cause. In Case 2, death during a fairground attraction raised the possibility of third-party liability; demonstration of a primary arrhythmogenic disorder excluded external causation. In Case 12, death occurred during occupational heat exposure, and negative genetic findings reinforced attribution to environmental and metabolic factors rather than inherited disease.

In this selected subgroup of complex forensic cases, integration of detailed pathological assessment with postmortem genetic testing provided incremental diagnostic value beyond conventional autopsy alone and was critical for accurate determination of both cause and manner of death.

## Discussion

Across heterogeneous forensic scenarios, integration of detailed pathological examination with postmortem genetic testing provided incremental diagnostic value beyond conventional autopsy alone. In autopsy-negative cases, genetic findings were decisive in establishing a natural cardiac cause of death, particularly in pediatric deaths and in events occurring during emotional stress or physical exertion. In cases with sub-diagnostic structural abnormalities suggestive of ACM, genetic results increased diagnostic confidence and supported interpretation of borderline myocardial changes as manifestations of inherited disease rather than incidental findings. Conversely, in cases characterized by inflammatory processes or uncommon structural lesions, negative genetic results contributed to delimiting the role of inherited cardiac disease and strengthened alternative pathological explanations.

### Diagnostic yield and concealed cardiomyopathy as a central challenge

In the present study, 12 cases were selected from a cohort of 343 medico-legal SUD autopsies performed over a 15-year period because conventional investigation was inconclusive or raised medico-legal concerns requiring clarification. This was therefore not a prevalence study but a focused analysis of complex forensic cases in which the integration of genetics had potential diagnostic and legal implications.

Within this selected subgroup, pathogenic or likely pathogenic variants were identified in 4 of 12 cases (33.3%), a yield consistent with previous reports showing actionable variants in 20–40% of unexplained sudden deaths and approximately 30% when cardiomyopathy is present at autopsy [12–15]. The observed yield should therefore be interpreted as reflecting case enrichment rather than overall population prevalence.

A central finding of our series is the distribution of clinically actionable variants across both channelopathy-associated and cardiomyopathy-associated genes. In two autopsy-negative pediatric cases, pathogenic variants in RYR2 and CALM2 provided a definitive diagnosis of primary electrical disease. These cases illustrate the well-established concept that structurally normal hearts do not exclude a lethal arrhythmogenic substrate and confirm that postmortem genetic testing is essential in pediatric and adolescent SUD [16–19].

Equally relevant, however, is the observation that in cases with sub-diagnostic structural findings, causal variants were predominantly located in cardiomyopathy-associated genes. The identification of a truncating FLNC variant and a homozygous PPP1R13L variant in cases with borderline or evolving structural phenotypes supports the concept of concealed cardiomyopathy, defined as malignant ventricular arrhythmias occurring before overt morphologic criteria are fully expressed [6, 8, 20]. This concept has gained increasing recognition in recent years and challenges the traditional dichotomy between “structurally normal” and “structurally abnormal” hearts. Subtle fibrofatty replacement, limited fibrosis, or mild hypertrophy may represent early or partial expression of inherited cardiomyopathy rather than incidental findings. Our data align with recent studies demonstrating that actionable variants in cardiomyopathy genes are frequently identified in autopsy-inconclusive cases and may be more common in the presence of subtle structural abnormalities than in completely normal hearts [6, 13, 15].

Traditionally, genetic testing in unexplained sudden death focused predominantly on inherited arrhythmia syndromes such as long QT syndrome or Brugada syndrome [15]. However, increasing evidence demonstrates that this approach underestimates the prevalence of cardiomyopathy-related variants in SCD. Genes such as *FLNC*,* TTN*,* LMNA*,* PKP2*,* DSP*, and *NKX2*.*5* are now recognized in autopsy-negative cases, often with subtle or sub-diagnostic pathological features [6, 21]. In fact, recent studies have shown that up to 70% of clinically actionable variants in autopsy-inconclusive cases are found in cardiomyopathy genes rather than channelopathy genes, and that these variants are four times more frequent in cases with sub-diagnostic structural abnormalities compared to structurally normal hearts [6, 13, 15]. Our findings support the systematic inclusion of both arrhythmia- and cardiomyopathy-associated genes in postmortem panels for SUD. Restrictive panels focused exclusively on channelopathies risk missing clinically actionable diagnoses in cases with subtle structural abnormalities.

### Variants of uncertain significance and ongoing challenges

Despite advances in sequencing technologies, a substantial proportion of SUD cases remain genetically unresolved, with 70–80% lacking definitive pathogenic variants even after broad testing [6, 12, 15]. VUS are particularly problematic in the post-mortem setting, where clinical correlation and segregation studies are often unavailable [11, 12]. In our experience, VUS were identified in 5 out of 12 cases (41.7%) in genes such as JUP, TXNRD2, GJA5, and LZTR1, which, although not definitively pathogenic, may represent candidates of emerging relevance [22–25]. For example, TXNRD2 is not currently considered a major ACM-associated gene; however, accumulating evidence suggests an emerging role in DCM [26, 27]. These findings illustrate both the potential of postmortem genetic investigation and the interpretative complexity it entails. For these reasons, hypothesis-free approaches (whole exome or genome sequencing without a clear suspicion) are not routinely recommended in forensic practice [6, 12, 15].

### Clinical, Preventive, and Medico-Legal Implications

Establishing a molecular diagnosis extends beyond determining the cause of death in the proband. Cascade family screening following sudden death has been shown to identify disease in 25–47% of screened relatives, particularly when multiple family members are evaluated [6, 28]. In our cohort, familial screening identified additional carriers, including clinically affected individuals who subsequently entered cardiological surveillance programs.

Identification of at-risk relatives enables targeted preventive strategies, including exercise restriction in ACM, pharmacological treatment, and prophylactic implantable cardioverter-defibrillator implantation in selected high-risk phenotypes [28, 29]. Given the age-dependent penetrance and variable expressivity of inherited cardiac diseases, longitudinal follow-up remains essential even in initially asymptomatic carriers.

One of the most distinctive contributions of postmortem genetic testing lies in its medico-legal impact. Several deaths in this series occurred under circumstances potentially suggestive of external causation, including physical confrontation, recreational activity, and occupational heat exposure. In such contexts, identification of an inherited cardiac substrate can prevent misclassification of death and avoid inappropriate attribution of criminal or civil liability [30, 31]. Conversely, negative genetic findings may reinforce attribution to inflammatory, environmental, or metabolic causes.

The legal significance of postmortem genetic evidence has been illustrated in internationally recognized judicial cases in which genetic findings fundamentally altered legal determinations. These examples underscore that postmortem genetic testing is not merely an academic adjunct but may serve as a safeguard against miscarriage of justice [32–34].

### Towards a multidisciplinary standard of care

The body of evidence supports the establishment of multidisciplinary teams including forensic pathologists, cardiologists, geneticists, pediatricians, psychologists, and genetic counselors [14]. Best practice requires systematic preservation of DNA samples (e.g., EDTA blood, spleen, liver, thymus, or fresh-frozen myocardium), use of broad next-generation sequencing panels, and careful interpretation of VUS following established international guidelines [4, 11]. This framework will facilitate the transition of post-mortem genetic testing from a research tool to a routine component of forensic and preventive medicine.

## Conclusion

Integration of systematic anatomopathological examination with postmortem genetic testing improves the accuracy of forensic investigation of sudden unexpected deaths and deepens understanding of inherited cardiac diseases. This combined approach supports appropriate medico-legal determinations and enables targeted preventive strategies in at-risk families.

## ANNEX I: Genetic Panels for the Study of Cardiomyopathies, Arrhythmias, and Sudden Cardiac Death

*Desmosomal genes (5 genes)*.

DSC2, DSG2, DSP, JUP, PKP2.

*Arrhythmogenic Cardiomyopathy Panel (21 genes)*.

CASQ2, CTNNA3, CTNNB1, DES, DSC2, DSG2, DSP, FLNC, JUP, LDB3, LMNA, PERP, PKP2, PKP4, PLN, PPP1R13L, RYR2, SCN5A, TGFB3, TMEM43, TTN.

*Panel for Cardiomyopathies*,* Arrhythmias*,* and Sudden Cardiac Death (218 genes)*.

A2ML1, AARS2, ABCC9, ACAD9, ACADVL, ACTA1, ACTC1, ACTN2, AGK, AGL, AGPAT2, AKAP9, ALMS1, ANK2, ANK3, ANKRD1, ANO5, ATP5E, ATPAF2, BAG3, BRAF, BSCL2, CACNA1C, CACNA1D, CACNA2D1, CACNB2, CALM1, CALM2, CALM3, CALR3, CAPN3, CASQ2, CAV3, CAVIN1, CAVIN4, CHRM2, COA5, COA6, COL7A1, COQ2, COX15, COX6B1, CRYAB, CSRP3, CTNNA3, CTNNB1, DES, DLD, DMD, DNAJC19, DNM1L, DOLK, DSC2, DSG2, DSP, DTNA, ELAC2, EMD, EYA4, FAH, FGF12, FHL1, FHL2, FHOD3, FKRP, FKTN, FLNC, FOXD4, FOXRED1, FXN, GAA, GATA4, GATA5, GATA6, GATAD1, GFM1, GJA1, GJA5, GLA, GLB1, GNPTAB, GPD1L, GREM2, GUSB, HCN4, HFE, HRAS, IDH2, ILK, IRX3, JPH2, JUP, KCNA5, KCND2, KCND3, KCNE1, KCNE2, KCNE3, KCNE5, KCNH2, KCNJ2, KCNJ5, KCNJ8, KCNK17, KCNK3, KCNQ1, KLF10, KRAS, LAMA2, LAMA4, LAMP2, LDB3, LDLR, LIAS, LMNA, LZTR1, MAP2K1, MAP2K2, MIB1, MLYCD, MRPL3, MRPL44, MRPS22, MTO1, MYBPC3, MYH11, MYH6, MYH7, MYL2, MYL3, MYLK2, MYOM1, MYOT, MYOZ2, MYPN, NEBL, NEXN, NF1, NKX2-5, NKX2-6, NNT, NOS1AP, NOTCH1, NPPA, NRAS, OBSCN, OBSL1, OPA3, PDHA1, PDLIM3, PERP, PHKA1, PITX2, PKP2, PKP4, PLN, PMM2, PPP1R13L, PRDM16, PRKAG2, PSEN1, PSEN2, PTPN11, RAF1, RANGRF, RASA2, RBM20, RIT1, RRAS, RYR2, SCN10A, SCN1B, SCN2B, SCN3B, SCN4B, SCN5A, SCO2, SDHA, SGCA, SGCB, SGCD, SHOC2, SLC22A5, SLC25A3, SLC25A4, SLMAP, SNTA1, SOS1, SOS2, SPEG, SPRED1, SURF1, SYNE1, SYNE2, TAZ, TBX20, TBX5, TCAP, TGFB3, TMEM43, TMEM70, TMPO, TNNC1, TNNI3, TNNI3K, TNNT2, TOR1AIP1, TPM1, TRDN, TRIM63, TRPM4, TSFM, TTN, TTR, TXNRD2, VCL, XK, ZFHX3.

*Panel for Cardiomyopathies*,* Arrhythmias*,* and Sudden Cardiac Death (251 genes)*.

A2ML1, AARS2, ABCC9, ACAD9, ACADVL, ACTA1, ACTC1, ACTN2, AGK, AGL, AGPAT2, AKAP9, AKT1, ALMS1, ALPK3, ANK2, ANK3, ANKRD1, ANO5, ATP5F1E, ATPAF2, BAG3, BRAF, BSCL2, C10orf71, CACNA1C, CACNA1D, CACNA2D1, CACNB2, CALM1, CALM2, CALM3, CALR, CALR3, CASQ2, CASZ1, CAV3, CAVIN1, CAVIN4, CBL, CDH2, CHRM2, COA5, COA6, COL7A1, COQ2, COX15, COX6B1, CRYAB, CSRP3, CTNNA1, CTNNA3, CTNNB1, DES, DLD, DMD, DNAJC19, DNM1L, DOLK, DSC2, DSG2, DSP, DTNA, ELAC2, EMD, EYA4, FAH, FBXO32, FGF12, FHL1, FHL2, FHOD3, FKRP, FKTN, FLNC, FOXRED1, FXN, GAA, GATA4, GATA5, GATA6, GATAD1, GFM1, GJA1, GJA5, GLA, GLB1, GNB2, GNPTAB, GPD1L, GREM2, GSK3B, GUSB, GYG1, HCN4, HFE, HRAS, IDH2, ILK, IRX3, ISM2, JARID2, JPH2, JUP, KAT6B, KCNA5, KCND2, KCND3, KCNE1, KCNE2, KCNE3, KCNE5, KCNH2, KCNJ2, KCNJ5, KCNJ8, KCNK17, KCNQ1, KLF10, KLHL24, KRAS, LAMA2, LAMA4, LAMP2, LDB3, LDLR, LIAS, LMNA, LMOD2, LZTR1, MAP2K1, MAP2K2, MAP3K8, MEF2C, MIB1, MLYCD, MRPL3, MRPL44, MRPS22, MTO1, MYBPC3, MYBPHL, MYH6, MYH7, MYL2, MYL3, MYLK2, MYOM1, MYOT, MYOZ2, MYPN, NEBL, NEXN, NF1, NKX2-5, NKX2-6, NNT, NONO, NOS1AP, NOTCH1, NPPA, NRAP, NRAS, OBSCN, OPA3, PDHA1, PDLIM3, PERP, PHKA1, PITX2, PKD2, PKP2, PKP4, PLN, PMM2, PPA2, PPCS, PPP1CB, PPP1R13L, PRDM16, PRKAG2, PSEN1, PSEN2, PTPN11, QRSL1, RAF1, RANGRF, RASA1, RASA2, RBM20, RBM24, RIT1, RRAS, RYR2, SCN10A, SCN1B, SCN2B, SCN3B, SCN4B, SCN5A, SCO2, SDHA, SGCA, SGCB, SGCD, SGCG, SHOC2, SLC22A5, SLC25A3, SLC25A4, SLMAP, SNTA1, SOS1, SOS2, SPEG, SPRED1, SPRY1, SURF1, SYNE1, SYNE2, SYNGAP1, TAZ, TBX20, TBX5, TCAP, TECRL, TGFB3, TMEM175, TMEM43, TMEM70, TMOD1, TNNC1, TNNI3, TNNI3K, TNNT2, TOR1AIP1, TPM1, TRDN, TRIM54, TRIM63, TRPM4, TSFM, TTN, TTR, TXNRD2, VCL, WISP1, WT1, XK, ZBTB17, ZFHX3.

*Panel for Cardiomyopathies*,* Arrhythmias*,* and Sudden Cardiac Death (288 genes)*.

A2ML1, AARS2, ABCC9, ACAD9, ACADVL, ACTA1, ACTC1, ACTN2, AGK, AGL, AGPAT2, AKAP9, AKT1, ALMS1, ALPK3, ANK2, ANKRD1, ANO5, ASNA1, ATP1A3, ATP5F1E, ATPAF2, BAG3, BRAF, BSCL2, BVES, C10orf71, CACNA1C, CACNA1D, CACNA2D1, CACNB2, CALM1, CALM2, CALM3, CALR, CALR3, CASQ2, CASZ1, CAV3, CAVIN1, CAVIN4, CBL, CDC25B, CDH2, CHRM2, COA5, COA6, COL7A1, COQ2, COX15, COX6B1, CRYAB, CSRP3, CTF1, CTNNA1, CTNNA3, CTNNB1, DES, DLD, DMD, DNAJC19, DNM1L, DOLK, DSC2, DSG2, DSP, DTNA, DYSF, ELAC2, EMD, EYA4, FAH, FBXO32, FGF12, FHL1, FHL2, FHOD3, FKRP, FKTN, FLNC, FNIP1, FOXD4, FOXRED1, FXN, GAA, GATA4, GATA5, GATA6, GATAD1, GFM1, GJA1, GJA5, GLA, GLB1, GNB2, GNPTAB, GPD1L, GREM2, GSK3B, GUSB, GYG1, GYS1, HAND1, HAND2, HCN4, HFE, HRAS, IDH2, ILK, IRX3, ISL1, JARID2, JPH2, JUP, KAT2B, KAT6B, KCNA5, KCND2, KCND3, KCNE1, KCNE2, KCNE3, KCNE5, KCNH2, KCNJ2, KCNJ3, KCNJ5, KCNJ8, KCNK17, KCNQ1, KLF10, KLF5, KLHL24, KRAS, LAMA2, LAMA4, LAMP2, LDB3, LEMD2, LIAS, LMNA, LMOD2, LRRC10, LZTR1, MAP2K1, MAP2K2, MAP3K8, MEF2C, MIB1, MLYCD, MRAS, MRPL3, MRPL44, MRPS22, MTO1, MYBPC3, MYBPHL, MYH6, MYH7, MYL2, MYL3, MYL4, MYLK2, MYLK3, MYOM1, MYOT, MYOZ2, MYPN, MYZAP, NAA10, NDUFB11, NEBL, NEXN, NF1, NKX2-5, NKX2-6, NNT, NONO, NOS1AP, NPPA, NRAP, NRAS, NUBPL, NUP155, OBSCN, P2RX7, PCCA, PCCB, PDHA1, PDLIM3, PDLIM5, PERP, PHKA1, PHKB, PITX2, PKD2, PKP2, PKP4, PLEKHM2, PLN, PMM2, POPDC2, PPA2, PPCS, PPP1CB, PPP1R13L, PRDM16, PRKAG2, PSEN1, PSEN2, PTPN11, QRSL1, RAF1, RANGRF, RASA1, RASA2, RBM20, RBM24, RIT1, RNF207, RPL3L, RRAD, RRAS, RYR2, SCN10A, SCN1B, SCN2B, SCN3B, SCN4A, SCN4B, SCN5A, SCO2, SDHA, SEMA3A, SGCA, SGCB, SGCD, SGCG, SHOC2, SHOX2, SLC22A5, SLC25A3, SLC25A4, SLC4A3, SLMAP, SNTA1, SOS1, SOS2, SPEG, SPRED1, SPRY1, SURF1, SYNE1, SYNE2, SYNGAP1, TAZ, TBX20, TBX5, TCAP, TECRL, TGFB3, TJP1, TLL2, TMEM43, TMEM70, TMOD1, TNNC1, TNNI3, TNNI3K, TNNT2, TOR1AIP1, TP63, TPM1, TRDN, TRIM54, TRIM63, TRPM4, TSFM, TTN, TTR, TUFM, TXNRD2, VARS2, VCL, WISP1, XK, ZBTB17, ZFHX3.
